# Perioperative management of patients undergoing combined heart-liver transplantation

**DOI:** 10.1186/cc13395

**Published:** 2014-03-17

**Authors:** DW Barbara, KH Rehfeldt, JK Heimbach, CB Rosen, RC Daly, JY Findlay

**Affiliations:** 1 Mayo Clinic, Rochester, MN, USA

## Introduction

Combined heart-liver transplantation (CHLT) is an uncommonly performed procedure for patients with coexisting cardiac and liver disease [1]. The purpose of this study was to examine and describe the perioperative management of patients undergoing CHLT.

## Methods

A retrospective review was performed of patients undergoing CHLT at our institution from 1999 to 2013.

## Results

Twenty-seven CHLTs were performed, with 4/27 including simultaneous kidney transplantation. Familial amyloidosis was the indication for 21 CHLTs (78%), and 12 of these explanted livers were used for domino transplantations. Nineteen patients (70%) were receiving inotropic infusions at the time of organ availability. The median preoperative MELD score was 12, and elevations in preoperative international normalized ratio were due to warfarin in all but one patient. Liver transplantation immediately preceded cardiac transplantation in 2/27 cases to reduce high-titer donor-specific antibodies. Venovenous bypass was utilized in 14 operations (52%) performed with the caval interposition liver transplantation approach, cardiopulmonary bypass during liver transplantation in two cases (7%), and no bypass in 11 operations (41%) performed with a caval sparing (piggyback) surgical technique. Postoperatively, the median duration of mechanical ventilation, ICU stay, and hospital stay until discharge were 1 day, 5.5 days, and 15 days, respectively. Transfusions in the first 48 hours following CHLT were not substantial in the majority of patients. One patient died within 30 days of CHLT.

## Conclusion

CHLT is a life-saving operation that is performed with relatively low mortality and can be successfully performed in select patients with congenital heart disease. Patients undergoing CHLT at our institution had relatively preserved hepatic function but limited cardiac function often requiring inotropic support. Cardiac transplantation typically precedes liver transplantation during CHLT given the decreased ischemic tolerance of the cardiac graft [2].However, liver transplantation prior to cardiac transplantation may serve to mitigate high-titer donor-specific antibodies. Various aforementioned operative approaches may be successfully utilized for the liver transplantation portion of these procedures. We attribute the favorable outcomes and perioperative courses to the multidisciplinary approach to care that CHLT patients receive at our institution.
